# Use of the surgical safety checklist in the operating room: Operating room nurses’ perspectives

**DOI:** 10.12669/pjms.35.3.29

**Published:** 2019

**Authors:** Oznur Gurlek Kisacik, Yeliz Cigerci

**Affiliations:** 1 *Dr. Oznur Gurlek Kisacik, Faculty of Health Science, Nursing Department, Afyonkarahisar University of Health Science, 03200 Afyonkarahisar/Turkey*; 2 *Dr. Yeliz Cigerci, Faculty of Health Science, Nursing Department, Afyonkarahisar University of Health Science, 03200 Afyonkarahisar/Turkey*

**Keywords:** Checklist, Operating room nurse, Patient safety, Surgery

## Abstract

**Objective:**

To determine the opinions of operating room nurses towards the Surgical Safety Checklist^TR^ (SSC^TR^) and to determine applications for using SSC^TR^ in operating rooms.

**Methods:**

This descriptive and cross-sectional study was conducted with 102 nurses working in the operating rooms of a state hospital and a university hospital in the Afyonkarahisar province. Descriptive statistics method were used for data analysis.

**Results:**

It was found that all operating room nurses knew the SSC^TR^ and that they had a positive opinion regarding the necessity of the SSC^TR^. However, most of the participants stated that the SSC^TR^ was not applied effectively in the operating room.

**Conclusion:**

The results obtained from the study show that changes focusing on the development of a culture of patient safety (PS) and team collaboration in operating rooms must be made in order to apply SSC^TR^ consistently and properly.

## INTRODUCTION

Although the main purpose is to save the life of the patient and improve the quality of life, practices which ignore patient safety (PS) during the surgical period and medical errors which occur are the main reasons for serious complications developed due to surgery.^1^ Operating rooms (ORs) are potential units that may threaten PS. More than half of unwanted events seen in hospitals are associated with the surgical care and at least half of these situations are preventable errors. It is reported that approximately 234 million people need surgical treatment for different medical reasons every year, and that 14% of these people experience an unwanted event.^2^

In 2008, the World Health Organization (WHO) initiated a campaign called “Safe Surgery Saves Lives” in order to draw attention to all these unwanted events resulting from surgical procedures and to improve the safety of surgery and consistency of surgical care based on the fact that at least half of surgical errors can be prevented with safe surgical practices. As an important part of this campaign, the WHO Safe Surgery Checklist (SSC) was developed in order to help improve teamwork among OR staff, to reduce mortality and complications in the perioperative process, and to ensure the consistent use of procedures for safe surgery.^3^ The results of the applying the checklist were evaluated with an international pilot study conducted in eight countries between 2007 and 2008. As a result of the study, it was determined that the rate of complication in surgical process decreased from 11% to 7% and that the mortality rate decreased from 1.5% to 0.8%.^4^ In 2009, the scope of the checklist in Turkey was expanded by the Head of Department of Quality and Accreditation, it was put into use with the name “Safe Surgery Checklist^TR^” (SSC^TR^). Since 2009, it has been used in hospitals as a mandatory standard for safe surgical practices in Turkey. However there are limited studies about its implementation.

Checklists used in the surgical process are among the most critical components of PS and emphasize subjects such as safe anesthesia and providing a safe airway, the right position and right region for surgery, prevention of infections and effective teamwork.^5^ The presence of consensus and collaboration among the team members in the OR about the use of SSC can provide more effective use of the checklist for preventing the complications and unwanted events that may occur due to the surgical process. Studies have shown that when the SSC and how and why it should be implemented was not well understood by the surgical team members then the importance given to the list decreased and disruptions were faced in its implementation.^6^ Operating room nurses (ORNs) can help to create necessary awareness among the members of the surgical team by acting as team leader in the process of applying the SSC.^7^

The purpose of this study was to examine the practices for the use of the SSC^TR^ in ORs by determining the opinions of those ORNs with important responsibilities in safe surgery practices. It is believed that the results of this study will help identify the problems in applying the ckecklist and the sharing of this information among surgical team members, will help surgical team members use the checklist more effectively by emphasizing the importance and benefits of SSC^TR^ and contribute to the development of PS in ORs.

## METHODS

Using a descriptive and cross-sectional research design, this study was carried out in the OR of a state hospital and a university hospital in Afyonkarahisar, in the Aegean region of Turkey between April and August 2017. The universe of the research consisted of a total 110 ORNs who had been working in the OR of the above mentioned hospitals for at least one month. Sample selection was not made and it was aimed to reach the whole universe of the study. During the study, eight nurses were excluded since five of them were on leave and three of them refused to participate. The study was completed with a total of 102 ORNs meeting the inclusion criteria. The response rate of the operating room nurses was 92.7%.

### Data Collection

A questionnaire consisting of 30 questions prepared by researchers after reviewing the related literature^1^,^6^,^8^-^11^ was used as a data collection tool. The questionnaire developed consisted of three parts. The first part of the questionnaire included eight questions examining the descriptive characteristics of nurses who participated in the research. In the second part, there were nine questions examining the surgical errors and type of errors encountered by the ORNs and their opinions about the process for reporting surgical errors. The last part of the questionnaire included 13 questions which were answered in the form of “yes”, “partially” and “no” and which examined the opinions of ORNs about the SSC^TR^ and its application in institutions. The content validity of the questionnaire was evaluated by five academic experts. At the end of the evaluation, the experts did not suggest any changes to the questionnaire. After this expert opinion had been received, a preliminary study was carried out with 10 ORNs. Questionnaires were delivered to the nurses by hand in an envelope so they could answer the questions themselves at an appropriate time.

### Ethical Considerations

Ethical approval for this study was obtained from the Clinical Research Ethics Committee, an University (approval number 2017/207) and written consent was obtained from the institutions where the research was conducted.

### Data Analysis

All analyses were performed using the SPSS statistical software package, version 22.0 (Armonk, NY: IBM Corp) Continuous variables were described using mean and standard deviations. Categorical variables were described using frequencies and percentages.

## RESULTS

It was determined that 89.2% of the nurses were female, 55.9% had undergraduate degrees, 44.1% had professional experience of 16 years or more, 30.4% had worked in an operating room for 5-10 years and their average age was 35.34 ± 7.07.

**Fig.1:**
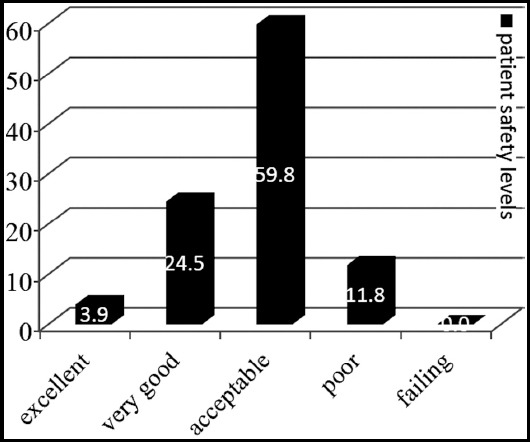
Patient safety levels in operating room reported by the nurses.

It was found that the majority of the ORNs (59.8%) rated the level of PS of the OR as “acceptable” and that only 3.9% of the nurses evaluated it as “excellent” (Fig.1).

While 71.6% of the nurses were found to have witnessed a surgical error in the OR, 42.5% stated that these errors had occurred within the last year. The vast majority (90.4%) of the errors encountered during the surgical process consisted of the operating physician “not conducting site marking”. In this study, 62.7% of the ORNs stated that they were aware of a system through which they could report medical errors in their institutions, although the majority (91.8%) was found not to have reported any surgical errors they witnessed in the OR (Table-I).

**Table-I:** Surgical errors encountered in ORs (n= 102).

Characteristics	Categories	n (%)
ORNs’ Experience of Encountering Surgical errors	Yes	73 (71.6)
	No	29 (28.4)
Surgical errors^§^	No site marking	66 (90.4)
	Not verifying patient identity	34 (46.6)
	Not transferring patients with health personnel	20 (27.4)
	Wrong site surgery	18 (24.7)
	Retained foreign bodies	16 (21.9)
	Medication error	16 (21.9)
	Patient fall	13 (17.8)
	Confusion or loss of pathology samples	11 (15.1)
	Not following infection control prevention procedures	5 (6.9)
	Blood and blood products transfusion error	1 (1.4)
	Development of burn in patient due to cautery/laser use	1 (1.4)
Status of reporting errors	Yes	6 (8.2)
	No	67 (91.8)

§ Multiple answers were given. OR: operatin room, ORNs: Opreating room nurses

All participants stated that they knew the SSC^TR^ and used it in their OR, while 80.4% had learned the SSC^TR^ in hospital in-service training. The majority of the participants stated that the SSC^TR^ was effective in ensuring PS and preventing complications due to surgery and 68.6% stated that it was effective in improving collaboration and communication within the OR team. In the study, only 11.8% of the nurses indicated that the SSC^TR^ was used according to its purpose, while the majority of the participants (82.4%) reported that the process of applying the checklist correctly in the OR was only “partially” carried out.

It was stated that the checklist was not implemented by a coordinator in the OR. On the other hand, the majority of the nurses (83.3%) stated that the SSC^TR^ was implemented by a circulating or scrub nurse and 64.7% of the nurses stated that they had problems while the checklist was being applied in their ORs. The problems stated by the ORNs and the factors affecting the correct use of the list are listed in Table-II. In this study, the main problem encountered during the application of SSC^TR^ was “surgeons’ unwillingness to wait for the control stages during the application of the checklist” (40%), and the second most common was “filling in the list without reading it out to the team” (30.7%). Situations such as intense working conditions in the OR (80.4%), disagreements within the OR team about the use and importance of the SSC^TR^ (69.6%) were also among the factors considered by the nurses to affect the correct application of the checklist (Table-II).

**Table-II:** Application of the SSC^TR^ in ORs (n= 102).

Characteristics	Categories	n (%)
Presence of a single coordinator responsible for application of the SSC^TR^	Yes	19 (18.6)
	No	83 (81.4)
Person applying the SSC^TR^	Circulating or scrub nurse	86 (83.3)
	Anesthesia technician	16 (16.7)
Duration required for application of the SSC^TR^	1-3 minutes	52 (51.0)
	3-5 minutes	42 (41.2)
	5-10 minutes	8 (7.9)
Status of facing problems in application of the SSC^TR^	Yes	66 (64.7)
	No	36 (35.3)
Problems faced during application of the SSC^TR§^ (N= 66)	Surgeons’ unwillingness to wait for control stages	26 (40.0)
	Filling in the SSC^TR^ individually without reading it to team	20 (30.7)
	Lack of time for the correct application of the SSC^TR^	9 (13.8)
	Lack of communication within the team	9 (13.8)
	Application of the SSC^TR^ solely as a mandatory procedure	9 (13.8)
	Not knowing how to use the SSC^TR^	4 (6.1)
	Not monitoring the correct use of the SSC^TR^	2 (3.0)
Factors affecting the use of the SSC^TR^ in ORs	Intense working conditions in the OR	82 (80.4)
	Disagreement within the OR team about the use and importance of the SSC^TR^	71 (69.6)
	Lack of knowledge about the use of the SSC^TR^	46 (45.1)
	Lack of information about the importance of the SSC^TR^	40 (48.0)

§ Multiple answers were given.

OR: operatin room, ORNs: Opreating room nurses

## DISCUSSION

The successful application of the SSC^TR^ is only possible with the awareness of surgical team about PS and their knowledge of the importance of using the checklist in surgery.^12^ Studies have shown that the use of the SSC^TR^ has a positive effect on the attitudes towards PS and helps to establish a PS culture in health institutions.^13^,^14^ In our study, the majority of the ORNs (59.8%) rated the level of PS in their ORs as “acceptable”. Similar to our findings, the results of other studies conducted in Turkey show that health workers were not very positive about the PS culture in their institutions and that they also evaluated the level of PS as “acceptable”.^15^ PS is a concept that has only begun to be developed in recent years in Turkey. The intense workload in hospitals and lack of a system for adequate personnel planning and PS may explain the fact that the ORNs in our study considered the level of PS to be only “acceptable”.

It has been reported an unwanted event occurs in OR at a rate of 47.7% to 50.3.^16^ In Turkey, approximately 43.6% of medical errors occur in OR.^8^ Similar to literature, in this study, the majority of the nurses (71.6%) had experienced a surgical error in OR. In our study, 90.4% of these errors were caused by the absence of site marking by the physician. Although the wrong patient/wrong site surgery is a condition preventable by following the standard procedures, it is still one of the most common surgical errors that are regularly reported globally.^17^ Two hundred and sixty of the 7734 surgical errors reported to the Safety Reporting System (SRS) in Turkey between 2016 and 2017 were the absence of surgical side/site marking.^18^ Studies investigating the causes of the wrong patient/wrong site surgery reveal that the main cause of these errors is a lack of communication between team members in OR.^10^,^17^ Surgical care requires the coordination of different disciplines in a complex environment at a critical time. The SSC^TR^ is an important tool in this. Our findings support the need for the use of the SSC^TR^ to improve collaboration and effective communication among surgical team members in order to prevent undesirable situations such as wrong patient/wrong site surgery in ORs.

We also found that the SSC^TR^ was known and used by all of the ORNs. In addition, it was determined that the majority of the nurses thought that the SSC^TR^ was effective in ensuring PS and preventing surgical complications. Similarly, in the study conducted by Abbasoğlu et al.^9^, it was reported that the majority of the nurses were aware of the SSC^TR^ and used the checklist. In another study, it was found that the more OR nurses were aware of the WHO’s SSC and its intended purpose compared to other surgical team members.^17^ Their awareness and positive attitude towards the purpose and importance of the SSC puts nurses in a leading position in using the checklist in a suitable manner in OR.

Active leadership is important for the successful application and sustainability of a checklist as well as for regular inspections and feedback.^6^ In the literature, it is reported that many hospitals give the responsibility of applying the SSC to the ORNs.^7^ In our study, it was found tha the checklist was mostly applied by circulating or scrub nurses. In another study,^9^ it was determined that the blank spaces in the SSC^TR^ that needed to be filled in by surgeons and anesthetists were mostly filled in by nurses. Similar to our findings, and as in the relevant studies,^6^,^8^,^9^,^11^ ORNs are those members of a team who have a high awareness of the importance and benefits of the SSC, who take responsibility, and who are more sensitive compared to surgeons and anesthetists about the factors preventing teamwork in the ORs. Additionally, these findings support the emphasis on the necessity for a nurse to be the SSC coordinator, as also stated by the WHO, in order to ensure safe surgical practices in the OR, to apply the checklist successfully and to increase all team members’ necessary awareness of the importance of the list. However, considering the factors such as the time that needs to be allocated for scrub nurses or circulating nurses to make the necessary preparations for the next operation, it is necessary that every institution understand the importance of giving support to help these nurses, who are known to be willing and enthusiastic about performing safe surgical procedures, apply the checklist effectively.

Another result we obtained in our study was that only 12 of the nurses stated that the checklist was correctly applied in OR. These results indicate that there are problems with the application of the SSC^TR^ and, as a result, there was not sufficient awareness of the positive effects of the SSC^TR^ in whole team members in the OR. The results of the study conducted by Candaş and Gürsoy^8^ are also similar to our findings. Similar results can be seen in most of the studies in which observance to SSC was assessed with the simultaneous observation and documentation. In yet another study^9^ it was determined that the checklist was used effectively; however, there were problems in filling in the list in the file review. In the study conducted by Levy and colleagues,^19^ it was determined that not all items on the checklist were fully applied in any of the cases. These results, which are similar to our findings, suggest that surgical team members have in an attitude that prevents the SSC being used effectively and sees the checklist as a mandatory standard documentary record which only needs to be filled out. This suggest the necessity of initiatives that focus on when and how to use the checklist. Moreover, in order for the checklist to be applied successfully, it is clear that the leadership of a coordinator who adopts a multidisciplinary team approach is also required.

Another finding in our study was that the nurses stated that they had problems during the application of the checklist in the OR. In a study, it was found that four out of five OR nurses had difficulty in applying the checklist.^20^ In a multicentered study, it was found that completing the checklist was time-consuming because of the heavy workload of personnel, that employees were not able to understand the benefits of the checklist and that some of the steps in the checklist were filled in without any assessment due to time constraints and only filled in in order to follow the hospital directives.^21^ In our study, the ORNs had similar problems to those found in the literature.

## CONCLUSION

This study provides information about the views and opinions of ORNs about the SSC^TR^, which is an important part of safe surgery practices, and its use in ORs. Although there is a high acceptance and adequate self-reported awareness of the SSC^TR^ among nurses, it is not always possible to implement it successfully. Another result of our study was that since SSC^TR^ is generally administered by ORNs, correctly applying the SSC^TR^ can be difficult for a nurse who needs to prepare for the next operation, especially considering the intense working conditions in OR. In addition, our findings suggest the necessity for a culture which ensures that surgical team members other than ORNs better understand the importance of the SSC^TR^. Training, effective leadership and communication is important for a successful implementation. Hospital administrations should establish initiatives to support and encourage SSC^TR^ use and to increase team communication and control. The awareness about PS should be increased and importance of SSC^TR^ use and application should be covered in educations.
